# Analysis of Factors Influencing the Efficiency of Catalysts Used in Waste PU Degradation

**DOI:** 10.3390/polym14245450

**Published:** 2022-12-13

**Authors:** Xiaohua Gu, Xiaoyao Wang, Tong Wang, Yanwei Zhu, Xinyu Guo, Siwen Liu, Shangwen Zhu, Yan Liu

**Affiliations:** 1 School of Energy and Building Environment, Guilin University of Aerospace Technology, Guilin 541004, China; 2 School of Material Science and Engineering, Qiqihar University, Qiqihar 161006, China; 3 State Key Laboratory for Modification of Chemical Fibers and Polymer Materials, College of Materials Science and Engineering, Donghua University, Shanghai 201620, China; 4 College of Chemistry and Chemical Engineering, Qiqihar University, Qiqihar 161006, China; 5 College of Innovative Material & Energy, Hubei University, Hubei 430062, China

**Keywords:** polyurethane waste, titanium glycol nanoparticles, catalyst, degradation and recycling, renewable polyurethane foam

## Abstract

Polyurethane (PU) is an indispensable part of people’s lives. With the development of polyurethane, the disposal of polyurethane waste has become a significant issue around the world. Conventional degradation catalysts have poor dispersion and low degradation efficiency when used in the process of solid degradation into liquid. Therefore, this paper innovatively adopts self-made core–shell nanoscale titanium catalysis, traditional alkali metal catalyst (KOH), and polyol to carry out the glycolysis of waste polyurethane (PU) pipeline foam. The homogenized nanoscale titanium catalyst coated with alcohol gel has an obvious core–shell structure. The alcohol gel not only protects the catalyst but also dissolves with the alcoholysis agent in the process of glycolysis and disperses more evenly into the alcoholysis agent to avoid the phenomenon of nanocatalyst agglomeration, so as to facilitate catalytic cracking without reducing catalyst activity. In this study, investigated and compared the production of renewable polyurethane foam via a one-step method based on use of a homogeneous core–shell nanostructured titanium catalyst vs. a traditional alkaline catalyst in terms of the properties of regenerated polyether polyols as well as of the foams produced from these polyols. The physicochemical properties of regenerated polyether polyols that were analyzed included viscosity, hydroxyl value, and average molecular weight. The regenerated polyurethane foams were characterized based on water absorption, TG, SEM, and thermal conductivity analyses. The results show that, when the addition of homogeneous titanium catalyst was T2 0.050 wt.%, the viscosity of regenerated polyether polyols was the lowest, at 5356.7 mPa·s, which was reduced by 9.97% compared with those obtained using the alkali metal catalyst (KOH). When the amount of titanium catalyst was T3 0.075 wt.%, the hard foam made of regenerated polyurethane prepared by the catalyst showed the best properties, with a compressive strength of 0.168 MPa, which is 4.76% higher than that of the foam prepared using KOH catalyst.

## 1. Introduction

Polyurethane (PU) materials first emerged in the 1930s in Germany [1], when Otto Bayer first discovered that new materials could be produced by reacting isocyanates with polyol compounds under certain conditions; these new materials were polyurethanes [2,3]. In the 1940s, Britain and the United States obtained the technology for the synthesis of polyurethane, and since then, polyurethane has been widely used all over the world. In recent years, with the rapid development of the construction, automobile, home appliance, textile, and other industries [4], the application fields and scale of polyurethane use have been expanding [5]. The huge production capacity of the polyurethane industry also means that a large amount of polyurethane solid waste is being generated, and the treatment of waste polyurethane foam is currently only in the theoretical stage. Physical methods for disposal such as landfill, piling, and incineration [6,7] cause huge secondary pollution to the environment, and chemical recycling methods such as alcoholysis [8,9], ammonolysis, pyrolysis [5,10,11], hydrolysis [12,13], and acidosis [14,15] are not amenable to industrialization. Therefore, the degradation and recycling of waste polyurethane foam remains a serious problem.

Izotz Amundarain et al. [16] studied recycling of industrial of polyurethane foam waste and synthesized regenerated rigid polyurethane foam by vacuum distillation and purification of the recovered polyols. The results show that the commercial polyols were partially replaced (up to 15 wt.%) by recycled polyols, which increased the reactivity of the synthesized polyurethane. Compared with traditional polyurethane foam, the foam prepared with recycled polyols had a lower density and smaller cell size. The addition of 10 wt.% recycled polyols resulted in a slight decrease in compressive properties and a significant increase in tensile strength and modulus values.

Catalysts play an important role in polyurethane degradation and recycling process as a bridge between grafted raw materials and products [17], and catalysts can significantly increase the rate of polyurethane degradation and improve productivity, whereas nanocatalysts have excellent properties [18,19] that can be exploited toward significantly increasing the rate of polyurethane degradation and improving productivity and product quality [20,21,22]. Polyurethane degradation catalysts mainly include inorganic salt compounds, organophosphorus oxides, and active alkali metals, which are easy to use and are used in numerous organic reactions [23]. The advantage of titanium-based catalysts include their higher activity [24]; titanium nanocatalysts, in particular, have the advantages of higher catalytic activity and ability to degrade PU [25,26].

In this paper, a homemade titanium nanocatalyst was used to explore the degradation of waste polyurethane pipe foam, which has the characteristics of high efficiency and high activity, good dispersion, few side reactions, nanoscale size, and high catalytic efficiency [27]. In addition, this catalyst has an obvious core–shell structure, and the surface is coated with a layer of alcohol gel. In the process of glycolysis, the alcohol gel on the surface is soluble in the alcoholysis agent, which can enable the catalyst to more evenly disperse in the alcoholysis environment [28]. Titanium nanocatalysts are widely used, low-cost, low-energy, stable, and environmentally friendly. Two different catalyst systems have been established with a homemade titanium nanocatalyst and a conventional alkali metal catalyst for the degradation and recovery of polyurethane pipe foam via glycolysis. Recycled polyether polyols were used to substitute for the conventional commercially available polyether polyols (up to 30 wt.%) in preparing renewable polyurethane foam via a one-step process. Because glycolysis uses a high-boiling point diol as an alcoholic solubilizer, and although the reaction temperature is relatively high and the energy consumption is high, the resulting renewable polyether polyol has a high hydroxyl value and can meet the required preparation conditions for rigid polyurethane foam [29,30]. The test data were analyzed by measuring the viscosity, hydroxyl value, and infrared spectra of the regenerated polyether polyol obtained from degradation as well as determining the properties of compression strength, water absorption, thermogravimetric weight, density, and thermal conductivity of the foams prepared using this regenerated product. The efficiency of the titanium nanosystem catalysts in catalytic degradation of waste polyurethane foam was investigated, and the resulting products were accordingly tested and characterized to explore the degradation mechanism and influencing factors.

In this study, the chemical recovery of waste polyurethane foam was carried out using the alcoholysis method, and the source of the regenerated polyether polyols was waste polyurethane foam. As they have certain rigid components (MDI/TDI/PAPI), the recovered polyether polyols also have highly rigid structures. With substitution (30 wt.%), the regenerated polyurethane foam prepared using conventional commercially available polyether polyols has a higher compressive strength and higher modulus than that prepared using conventional foams. The considerable amount of replacement not only reduces the cost of the production process but also realizes global recycling strategy concepts of turning plastic waste into useful material and industrial waste recycling [31].

## 2. Materials and Methods

### 2.1. Materials and Reagents

The main reagents used in this experiment for recycling waste polyurethane are as follows: waste polyurethane direct buried pipe rigid foam, Daqing direct buried pipe foam, refrigerator waste foam; ethylene glycol, Tianjin Chemical Reagent No.1 Factory, analytically pure (AR); propylene glycol, Tianjin Chemical Reagent No.1 Factory, analytically pure (AR); butylene glycol, Tianjin Kaitong Chemical Reagent Co. Chemical Reagent Company (Tianjin, China), analytically pure (AR); ethanolamine, Langfang Quanzhen Chemical Co., Ltd., (Langfang China), AR; potassium hydroxide, alkaline catalyst, Tianjin Tianli Chemical Reagent Co., (Tianjin China), Tetrabutyl titanate, Nanjing Youpu Chemical Co., Ltd., (Nanjing, China), AR; anhydrous ethanol, deionized water.

The main reagents used in the foaming experiment of recycled polyether polyol from degraded waste polyurethane are as follows: silicone oil stabilizer, Guangzhou Feirui Chemical Co., (Guangzhou, China), Triethanolamine, Shanghai Demao Chemical Co., Ltd., (Shanghai, China); tin solution, Beijing Zhongnuo Taian Technology Co., Ltd. (Beijing, China); blowing agent HCFC-141b, Shenzhen Huachang Chemical Co., Ltd. (Shenzhen, China); polyether 4110, Shandong Lianhao Yao New Material Co Ltd., (Shouguang, China,. Triethanolamine, Shanghai Demao Chemical Co., Ltd., (Shanghai China); tin solution, Beijing Zhongnuo Taian Technology Co., Ltd. (Beijing, China); blowing agent HCFC-141b, Shenzhen Huachang Chemical Co., Ltd. (Shenzhen, China); polyether 4110, Shandong Lianhao Yao New Material Co.

### 2.2. Preparation of Titanium Nanosystem Catalysts

**Figure 1:**
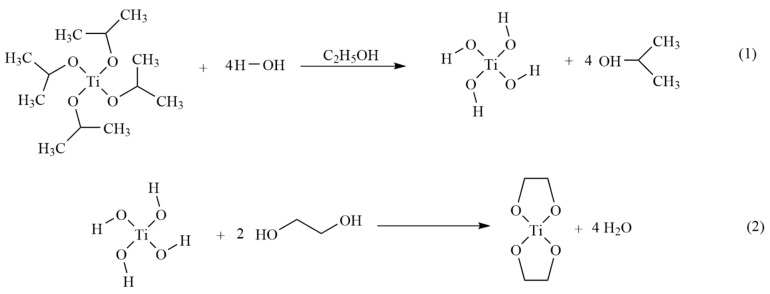
Synthesis route of titanium ethylene glycol nanocatalyst.

In this experiment, 43.2 g (0.152 mol) of tetrabutyl titanate and 260 g (5.643 mol) of anhydrous ethanol were added into a 2 L three-necked flask, and a mixture of 13.5 g of deionized water and 43 g of anhydrous ethanol was slowly added dropwise via a dosing tube under the conditions of incubation in an oil bath at 30 °C and high-speed stirring, and the addition was controlled such that the desired amount was added dropwise over a period of about 3 h. The crude intermediate product was obtained, which was then refluxed at the appropriate temperature for 3 h and added to ethylene glycol followed by resting; finally, vacuum distillation was carried out at a certain temperature to obtain the colorless or light yellow titanium nano catalyst liquid. The route for synthesis of the titanium nanodiol catalyst is shown in Figure 1 below.

### 2.3. Recycling of Waste Polyurethane

According to our previous research, the optimal three-component alcoholic solubilizer ratio for degrading polyurethane foam is ethylene glycol (EG)/propylene glycol (PDO)/butylene glycol (BDO) = 35:35:30. The recycled waste polyurethane foam was crushed into 2–10 mm polyurethane foam chips using a shredder, and then 35 g of ethylene glycol, 35 g of propylene glycol, and 30 g of butylene glycol (total mass 100 g), as well as different ratios of KOH/titanium glycol catalyst (T1: 0.025 wt.%; T2: 0.05 wt.%; T3: 0.075 wt.%; T4: 0.10 wt.%; T5: 0.20 wt.%), were added into the glass reactor at 90 °C, and the catalyst was left to dissolve completely at this temperature (the temperature was raised from room temperature to 90 °C after the liquid titanium glycol was added). After the catalyst had completely dissolved, the alcoholic solubilizer was added to the pre-crushed waste polyurethane foam scraps at a mass ratio of 1:1 for further reaction, and the temperature of the reaction at this stage was increased to 180 °C. The reaction ran for 3–5 h to allow complete degradation of the waste polyurethane scraps and to obtain recycled polyether polyol, which was cooled to room temperature and transferred to a disposable plastic cup for collection. The alcoholytic agent breaks the isocyanate in the polyurethane chain segment, thus generating relatively short chain segments, which produces recycled polyether polyols that can be further recycled for use.

### 2.4. Preparation of Renewable Polyurethane Foams

**Figure 2:**
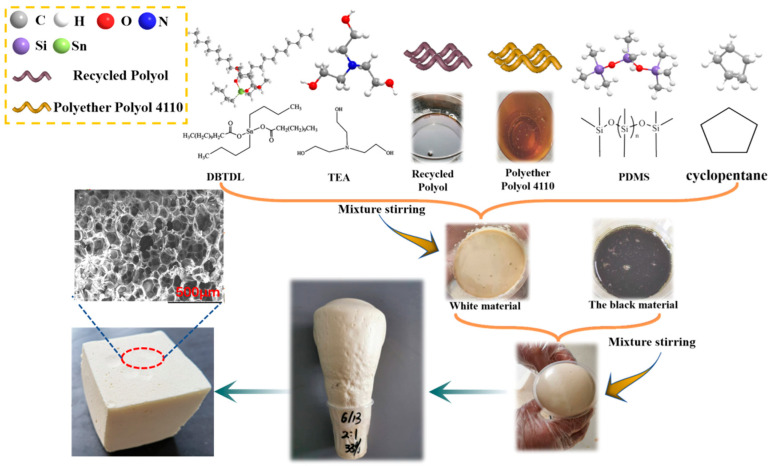
Flow of foaming process used for replacing some commercially available polyether polyols with recycled polyether polyols.

The recycled polyurethane rigid foam was prepared using a one-step method, and the specific operation formula is shown in Table 1. In the foaming experiment, the surfactant (PDMS), foaming agent (HCFC-141b), and foaming catalyst (TEA, DBTDL) were added to the disposable plastic cup containing regenerated polyether polyol in a given proportion and stirred well at 1000 rpm/min, after which a certain amount of black material (PAPI) was added and stirred at the same rate. When the wall of the disposable plastic cup felt obviously warmer, we stopped stirring and waited while foaming continued to obtain the recycled polyurethane rigid foam. The resulting recycled polyurethane rigid foam was crosslinked and cured at 60 °C for 20 min and then cured at room temperature for more than 24 h. The samples were then characterized. Figure 2 shows the process flow of the foaming of renewable polyether polyol, with replacing part of the commercially available polyether polyol.

### 2.5. Performance Test and Structural Characterization of Polyurethane Rigid Foam

Viscosity analysis: An NDJ-5S digital viscometer was used for viscosity testing; a number of degradation products were taken and placed in the container while a suitable rotor was selected as well as a suitable rotational speed, and viscosity testing was carried out at 25 °C.

Compression strength test: We referred to the GB/T8813-2008 test standard—we took a sample size of 50 mm × 50 mm × 50 mm and used an EFS-24RE universal testing machine for the compression strength test.

Water absorption test: We referred to the GB/T8810-1988 test standard—the sample size was 50 mm × 10 mm × 10 mm, and we used distilled water to determine the water absorption by weighing the mass of the sample before and after immersion to calculate its water absorption.

Hydroxyl value determination: We referred to the GB/T12008.3-2009 standard, taking appropriate amounts of oligomeric regenerated polyether polyol in a 10 mL conical flask, and used the ester anhydride method with pyridine for hydroxyl value determination.

Thermal conductivity analysis: Referring to the QB/T3806-1999 standard, the sample size was 200 mm × 200 mm × 20 mm, and we used the FEHC-S thermal conductivity tester of Changzhou Hua’ao Instrument Manufacturing Co Ltd., (Changzhou, China).

SEM analysis: We cut the regenerated polyurethane rigid foam specimens into appropriately thin slices (without squeezing) and then used SEM to observe the microstructure of the bubble pores of the foam, magnified 20 times, observing the clear, complete and uniform area of the bubble pore structure and recording the observation results.

FT-IR test: GR-285 IR produced by Dalian Precision Scientific Instruments Co. Ltd., (Dalian, China). was used to analyze the structure of the foam samples, and the samples were formed by the KBr pressing method; the test wavelength range was 500–4000 cm^−1^.

TG analysis: TG analysis was performed using TG under a nitrogen atmosphere at a rate of 30 °C/min from room temperature (25 °C) to 500 °C. The gas flow rate was set at 50 mL/min, the carrier gas was air, and alumina was used as the reference object.

Determination of molecular weight distribution coefficient: The molecular weight distribution of the regenerated polyether polyol was determined by gel permeation chromatography (GPC). Measurements were performed using a thermal scientific chromatograph equipped with an isocratic DionexUltra3000 pump and a RefrtoMax521 refractive index detector. Separations were performed in four Phenomenex Phenogel GPC columns with a separation temperature of 30 °C, a particle size of 5 µm, and different porosities of 105, 103, 102, and 50 while placed in an ultimate thermostatic column at 3000 °C. The mobile phase was tetrahydrofuran (THF) set at a flow rate of 1 mL·min^−1^. The samples were first dissolved in N,N-dimethylformamide(DMF) followed by 1.6 wt.% THF, filtered through a nylon filter with a pore size of 2 mm, and then prepared for use.

TEM test: The prepared ethylene glycol titanium samples were diluted and dispersed in anhydrous ethanol solution and then ultrasonically stirred using ultrasonic equipment to obtain a uniform suspended liquid. The drops were placed on a copper net with a supporting film, and the dispersion was tested after volatilizing in a dry environment.

## 3. Results and Discussion

### 3.1. Mechanism of PU Degradation by Titanium Nanosystem Catalysts

**Figure 3:**
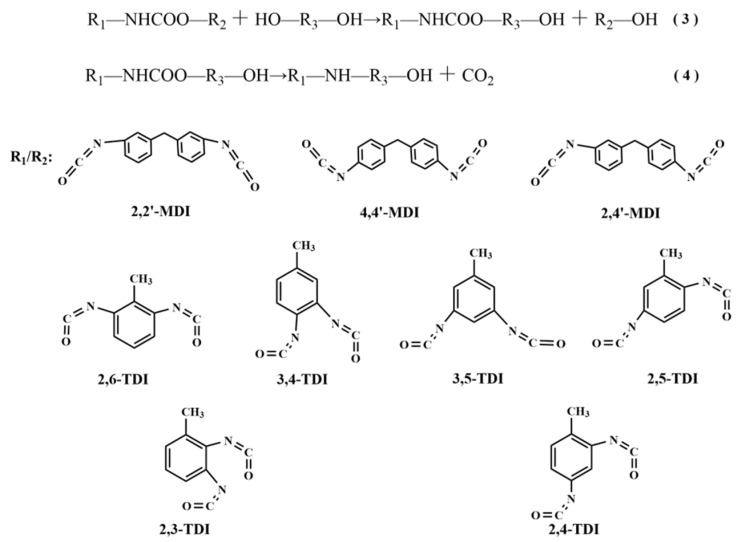
Mechanism of polyurethane foam degradation catalyzed by titanium ethylene glycol nanoparticles.

Waste polyurethane rigid foam is mainly composed of carbamate bonds. Under certain temperature conditions, the carbamate bonds are broken under the joint action of alcoholic solvents and catalysts, then replaced by alcoholic solvent chain segments to generate renewable polyether polyols, and the specific main reaction mechanism is shown in Figure 3.

**Figure 4:**
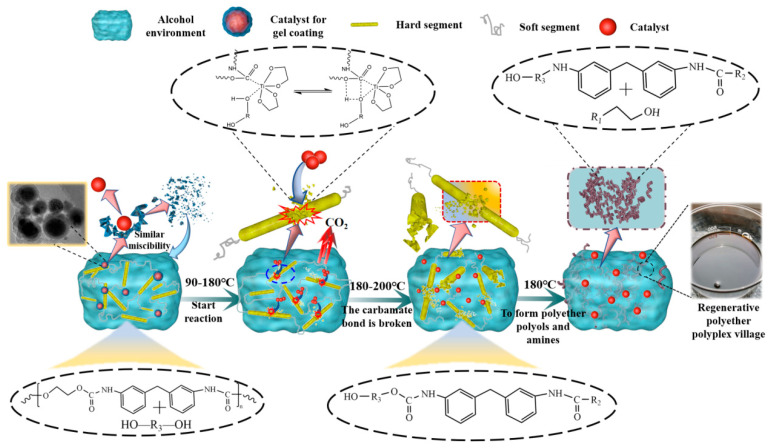
Mechanism of the titanium glycol-catalyzed degradation of 2,2′-diphenylmethane diisocyanate (MDI)-type polyurethane hard foam.

Equation (3) in Figure 3 shows the breakage of the carbamate bond in the waste polyurethane rigid foam under the action of the alcoholytic agent, and Equation (4) in Figure 3 shows the process of the further reaction to produce carbon dioxide. Then, the catalytic degradation of polyurethane hard foam synthesized with 2,2′-diphenylmethane diisocyanate (MDI) as the main raw material was carried out, and the mechanism is shown in Figure 4.

Ethylene glycol titanium was prepared by adding a large amount of ethylene glycol, which led to a complexation reaction in the system, resulting in an ethylene glycol titanium catalyst with an outer layer covered with an alcohol gel. The alcoholytic agent used in this experiment was ethylene glycol (EG)/propylene glycol (PDO)/butylene glycol (BDO) = 35:35:30. When the ethylene glycol contained in the alcoholic solubilizer encountered the ethylene glycol titanium catalyst, the alcoholic gel covering the outer layer of the catalyst acted similarly to the alcoholic solubilizer in that it steadily released the catalyst and promoted good dispersion of the titanium nanoparticles without agglomeration, maximizing the catalyst activity through release of the ethylene glycol titanium catalyst in the alcoholic solubilizer. Following this, the crushed polyurethane foam was added. When the polyurethane foam met the alcoholizing agent, the alcoholizing agent and catalyst were absorbed into the foam. As the temperature increases, the titanium glycol catalyst attached to the polyurethane foam skeleton catalyzes the cracking of the polyurethane rigid section at the appropriate temperature, which eventually completely breaks the rigid section of the polyurethane foam to produce free small-molecule polyether polyols and amines by substitution with the small-molecule alcohols in the alcoholytic agent.

**Figure 5:**
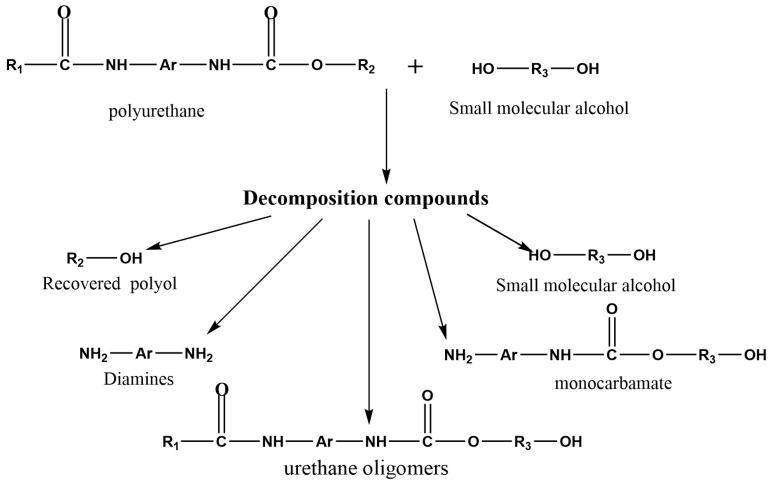
Outline of possible products obtained from the alcoholysis process.

The mechanism polyurethane alcoholysis degradation usually generates the following products [16] shown in Figure 5.

**Figure 6:**
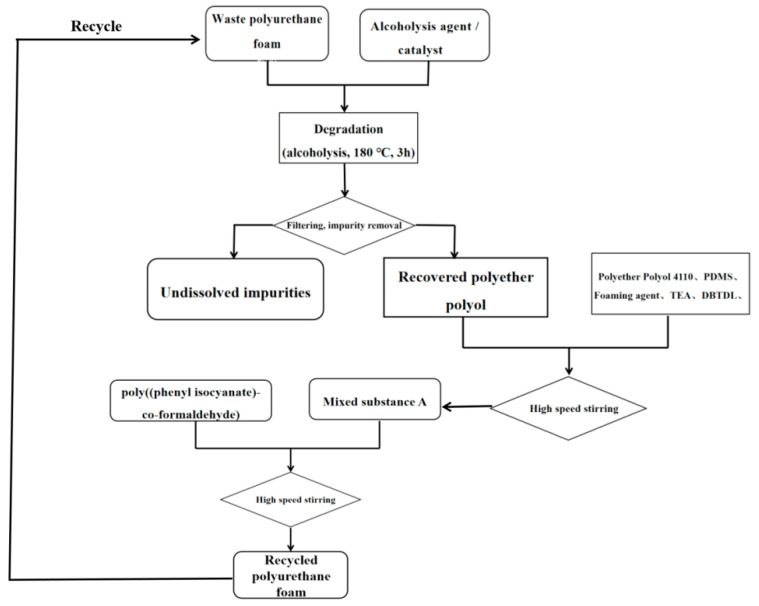
Flow chart of degradation and foaming of waste polyurethane in the production of recycled polyurethane foam.

The flow of the process of recycling waste polyurethane foam, through its degradation by alcoholysis and subsequent use of the recycled polyether polyols to replace commercially available polyether polyols in the production of recycled polyurethane foam, is shown in the Figure 6.

### 3.2. Infrared Spectroscopy Analysis of the Nanosized Ethylene Glycol Titanium Catalyst

**Figure 7:**
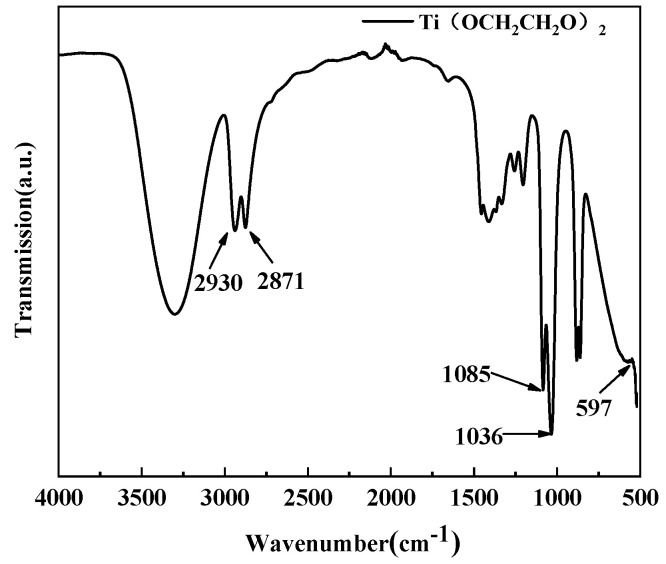
Infrared spectroscopy of the nano-ethylene glycol titanium catalyst.

Figure 7 shows FT−IR characterization of the homemade titanium ethylene glycol nanocatalyst used in this experiment shows that the peaks in the absorption band at 597 cm^−1^ are mainly Ti-O bond stretching vibrations; the peaks at 2930 and 2871 cm^−1^ are characteristic absorption peaks of sp^3^ C-H stretching vibrations; the peaks at 1085 and 1036 cm^−1^ are the peaks of C-H in-plane bending vibrations and C-O stretching vibration, respectively [32]. The characterization shown in the above IR spectrograms is consistent with the structure of the homemade titania catalyst.

### 3.3. Transmission Electron Microscopic Analysis of Nano−Ethylene Glycol Titanium Catalyst

**Figure 8:**
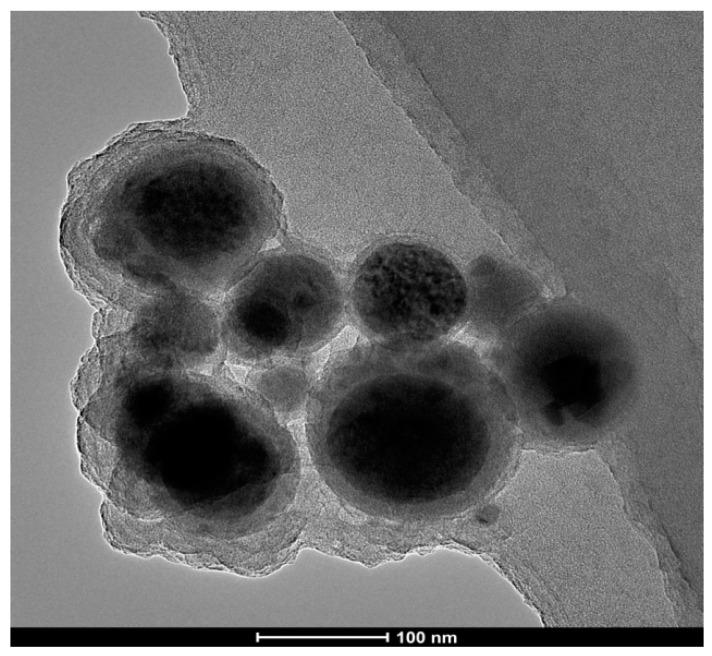
Transmission electron microscope image of nano−ethylene glycol titanium catalyst.

Figure 8 shows a transmission electron micrograph of the nano-ethylene glycol titanium catalyst, from which it can be seen that the catalyst particles are all around 100 nm, nearly spherical [11,33,34,35], and present an obvious core–shell structure. Therefore, it has the unique surface effects of nanomaterials, and the small particle size of the ethylene glycol titanium catalyst increases the number of titanium atoms on its surface, meaning the surface area and surface tension become larger, which leads to higher chemical activity. Therefore, due to its high activity and high catalytic efficiency, only a small amount of catalyst needs be added in actual production to achieve the catalytic purpose; there is thus no need to consider the problem of removing the catalyst from the later products, as it will not affect their performance.

### 3.4. Infrared Spectroscopy Analysis of Recycled Polyether Polyols

**Figure 9:**
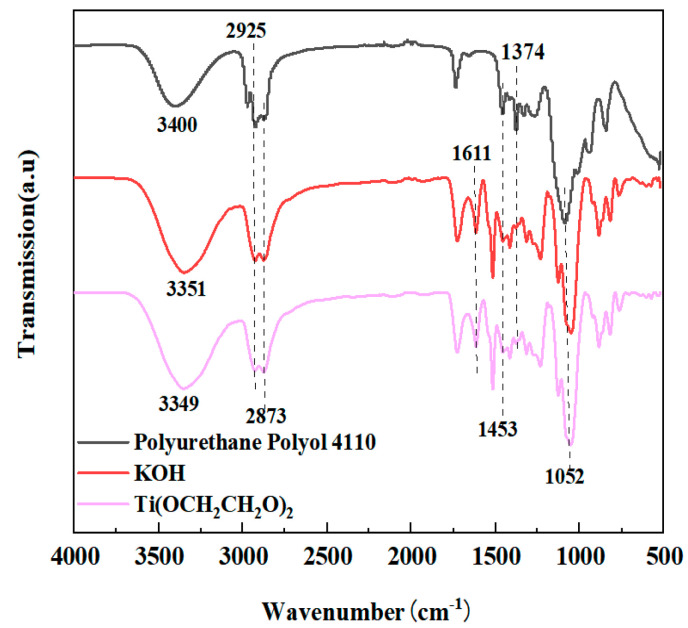
IR spectra of recycled polyol and polyether polyol 4110 recovered from different catalysts.

Figure 9 shows a comparison of the IR characterization of the regenerated polyether polyol obtained after alcohol degradation of the used polyurethane hard foam with a conventional alkali metal catalyst KOH, a homemade ethylene glycol titanium Ti(OCH_2_CH_2_O)_2_ catalyst, and polyether polyol 4110 from polyurethane feedstock, respectively, and the results are shown in Figure 8. It can be seen from the figure that the peaks of the products obtained after the degradation of the polyurethane hard foam with two different catalysts are basically similar to the characteristic peak of polyether polyol 4110 in polyurethane feedstock, showing a distinct and intense peak at 3351 cm^−1^, which is the characteristic absorption peak of alcohol hydroxyl group (–OH) [36,37]. The peaks at 2925 and 2873 cm^−1^ correspond to sp C−H stretching vibration; through comparison with the spectrum for polyether 4110 IR, a clear absorption peak appears at 1611 cm^−1^, which is a phenyl broadband peak [38]. Polyether 4110 itself does not have a benzene ring, and the phenyl peak appears in the degraded material because the degraded material is obtained from the recycling of waste polyurethane, and polyurethane is synthesized from MDI/TDI as raw material, so the spectrum of the obtained renewable polyether polyol shows a phenyl peak. The isocyanate heel (N=C=O) in the synthetic polyurethane foam black material (PAPI) did not appear at 2270 cm^−1^, confirming the successful degradation of waste polyurethane foam via the alcoholysis reaction [39]. The characteristic C−H bending vibration peaks of polyether polyol are observed at 1453 and 1374 cm^−1^ [40], and the typical peak for characteristic C–O–C vibration in polyether polyol is at 1052 cm^−1^ [41]. From the above characteristic peaks, it can be seen that the used polyurethane rigid foam was successfully degraded into a small-molecule polyol similar to polyether polyol 4110 under the actions of alcoholic solvents and catalysts, and the two are chemically similar, as can be inferred from the characteristic functional groups, so the regenerated polyether polyol obtained from degradation can be used to replace the polyether polyol 4110 in polyurethane raw materials for foaming.

### 3.5. The Viscosity of Regenerated Polyether Polyol and the Compressive Strength of Regenerated Polyurethane Foam

**Figure 10:**
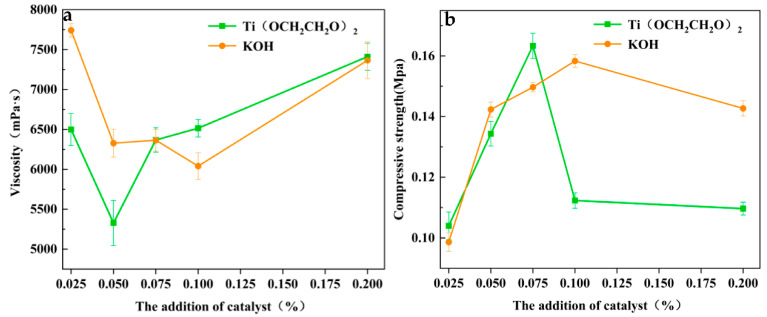
Effects of different catalytic systems on the viscosity of regenerated polyether polyol (**a**) and the compressive strength of regenerated PU (**b**).

Other experimental conditions were fixed, and the used polyurethane direct buried pipe rigid foam was degraded with an alkali metal KOH catalyst and homemade nanosized ethylene glycol titanium catalyst in appropriate alcoholic solvents. The viscosity of degraded products was then assessed using an NDJ-5S digital viscometer. The test results are shown in Figure 10a. This indicates that when only added in a small amount, the traditional catalysts do not degrade the used polyurethane foam sufficiently due to their low activity, whereas the glycol titanium catalysts have a large specific surface area due to their nanometer size and surface effect. The viscosity of the glycol titanium catalyst system is lower because of its larger specific surface area and higher catalytic effect in the reaction system; when more catalyst is added (>0.050%), the viscosity of the traditional alkali metal catalyst is lower than that of the glycol titanium catalyst system because of the increase in the amount added, and the catalytic efficiency of the reaction system is thus improved. When the addition amount is increased, the waste polyurethane foam in the reaction system will be degraded into smaller short-chain small molecules, which will be agglomerated later, thus leading to the steep increase in the viscosity of the ethylene glycol titanium catalyst [42];the viscosity is, however, higher than that of the traditional alkali metal catalyst. Following this, foaming experiments were conducted on the regenerated polyether polyol, and the compression strength of the regenerated polyurethane foam was tested via an EFS-24RE universal testing machine, as shown in Figure 10b. This showed that the strength of the foams prepared by the degradation of different catalyst systems increase and then decrease with the increase in the amount of added catalyst. Generally speaking, the compression strength of regenerated polyurethane foam decreases with the increase in the amount of recycled polyether polyol replaced [43]. Therefore, after the adjustment of the foaming formula, the replacement amount was finally increased to 30 wt.% under the premise of the normal use of the regenerated polyurethane foam.

The good interfacial compatibility of titanium glycol and polyurethane leads to a mechanism under which the polyurethane and the catalyst have satisfactory compatibility, resulting in the good interfacial compatibility of the catalyst of titanium glycol in the solvent used in the process of degradation into small molecules. This ensures the good dispersion of the small titanium glycol molecules in the alcoholic solubilizer, allowing the highest possible effect and activity of the catalyst and, therefore, complete degradation of polyurethane. A polyether polyol with satisfactory properties is obtained, so the resulting regenerated polyether polyol also has satisfactory properties, and the polymer foam obtained after the application of re-polymerization will have strong compression properties. The titanium ethylene glycol catalyst yields foams with higher compression strengths when prepared from degraded polyether polyol compared with the alkaline earth catalyst.

### 3.6. Hydroxy Values of Regenerated Polyether Polyols

The hydroxyl values of the regenerated polyether polyols recovered from the used polyurethane foam degraded under different catalyst systems were determined using the ester-anhydride method with pyridine under the same experimental conditions.

**Figure 11:**
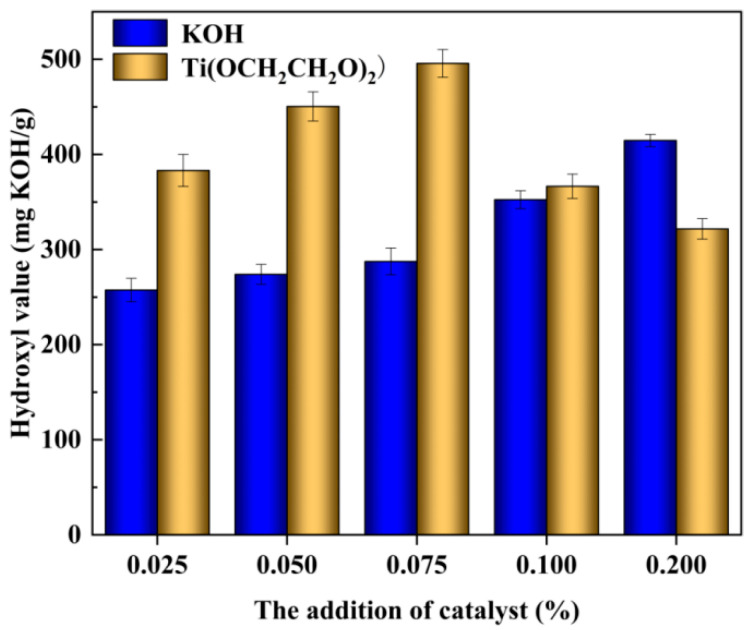
Determination of the hydroxyl value of the regenerated polyether polyol under different catalytic systems.

As can be seen from Figure 11, the hydroxyl value of the used polyurethane rigid foam recovered using the traditional alkali metal KOH catalyst under different catalytic systems showed an increasing trend with the addition of the catalyst, because the activity of the traditional catalyst is weak compared with that of the ethylene glycol titanium catalyst, and a small amount of the traditional alkali metal catalyst cannot sufficiently break the carbamate bonds in the used polyurethane foam, resulting in a system with more end-hydroxyl or branched hydroxyl groups and a lower hydroxyl value. As the amount of added traditional catalyst is increased, the catalytic efficiency of the system increases, and there is increased efficiency of carbamate bond breaking in the waste polyurethane foam such that the polyurethane chain segments in the system are degraded into many relatively short-chain segments with end-hydroxyl or branched hydroxyl groups [44], which causes the hydroxyl value to increase. For the ethylene glycol titanium catalyst, when the amount of catalyst is increased to a certain value (≥0.100%), although the system reaction is vigorous, the hydroxyl value is significantly reduced because the excessive catalytic activity of ethylene glycol titanium, which leads to the polyurethane foam carbamate chain segment being crushed and fractured and then rejoined, resulting in a reduction in the hydroxyl value. This gives rise to a viscosity similar to that of the regenerated polyether polyols mentioned above.

### 3.7. Thermal Conductivity and Molecular Weight Distribution Coefficient of Recycled Polyether Polyols

**Figure 12:**
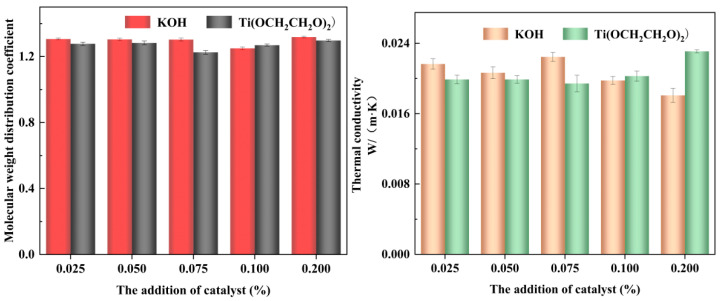
Determination of the molecular weight distribution coefficient of the regenerated polyether polyol and the thermal conductivity of the regenerated polyurethane foam under different catalytic systems.

From the Figure 12, it can be seen that the molecular weight distribution coefficients of the renewable environmentally friendly polyether polyols obtained by the degradation of different catalytic systems do not differ significantly. It can be seen that the waste polyurethane was successfully degraded under different catalyst systems, and the molecular weight distribution coefficients of the molecular products in the degraded renewable and environmentally friendly polyether polyols were relatively uniform and similar to those of the commercially available polyether 4110.

From the macroscopic point of view, the value of thermal conductivity is closely related to the integrity of the polyurethane foam’s bubble pores, the degree of fit of the bubble pore wall, and the skeleton thickness. From the microscopic point of view, the value of thermal conductivity is closely related to the selection and proportion of alcoholic solvents, catalyst activity, temperature, degradation time, degree of urethane bond breakage during degradation, amount of foaming agent added during foaming, amount of catalyst used in foaming. All macroscopic and microscopic factors have either direct or indirect influences on the polyurethane pore shape, which further affect the thermal insulation performance of polyurethane foam and thus cause the thermal conductivity to vary. Generally speaking, more complete degradation of the used polyurethane foam is achieved under appropriate pressure, temperature, and time conditions, and this leads to the more thorough urethane bond breakage and more uniform molecular weight distributions of the generated short-chain materials, such as the polyether polyol recovered from the degradation material, which is more favorable for the foaming reaction. Therefore, the more thorough the reaction of the degraded waste polyurethane foam, and the higher the amount of appropriate foaming agent and catalyst added during the foaming process, the more complete the pores of the regenerated polyurethane foam, the closer the pore walls are to each other, and the stronger the skeleton. The polyurethane foam generated under the optimal conditions will achieve the best heat preservation performance.

Polyurethane foam achieves better thermal insulation performance and a lower thermal conductivity because the movement of gas entering the foam is obstructed, and the complete pores and thick pore walls of the foam prevent the convective heat transfer enabled by air flow. These pore walls act as a barrier to prevent the air from passing quickly through the foam, thus causing the gas to stay in the foam for a much longer time and thus achieving the effect of heat insulation. Therefore, the bubble pore integrity and pore wall thickness of the foam directly affect its thermal insulation and thermal conductivity properties [45].

From Table 2, it can be seen that the thermal conductivity values of various recycled Ti(OCH_2_CH_2_O)_2_ polyols are relatively low under different degradation systems but essentially similar and fully comply with the national standard, indicating that they are suitable for use in actual production.

### 3.8. TG Test of Regenerated Polyurethane Hard Foam

**Figure 13:**
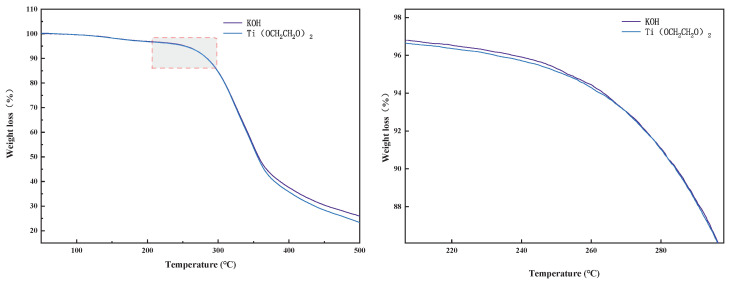
TG analysis of the regenerated polyurethane foam.

Figure 13 shows the TG analysis of the satisfactory regenerated polyurethane hard foam products with different additions of KOH and Ti(OCH_2_CH_2_O)_2_ for two different catalyst systems. The temperature range of this test is from 25 °C (room temperature) to 500 °C. It can be seen from the figure that the starting decomposition temperatures of the two regenerated polyurethane foams are around 240 °C, and the reaction ends at 500 °C.

Thermogravimetric weight can reflect the relationship between the polyurethane foam mass and temperature. The decomposition of polyurethane foam is generally divided into three stages [23]: the first stage is the evaporation of free water from the surface of the polyurethane foam, which is usually not evident in this stage because of the low free water content in polyurethane foam itself; the second stage is the breakage of the hard segment (HS) in the polyurethane foam, which contains structural units composed of isocyanates as well as aromatics in polyurethane foam; the third stage is the fracture of the soft satin (SS), which contains structural units, such as aliphatic polyethers, in the polyurethane foam [37]. Zooming in on Figure 11, it can be seen that the onset of weight loss of the regenerated polyurethane foams prepared by degradation with the two different catalytic systems is, more precisely, at around 243 °C. The regenerated polyurethane foams made by degradation with the alkali metal catalyst (KOH) are the first to exhibit weight loss, with a maximum thermal weight loss rate around 330 °C. The regenerated polyurethane foam prepared via degradation with the new titanium catalyst (Ti(OCH_2_CH_2_O)_2_) initially lost weight slowly, and the maximum thermal weight loss rate was around 340 °C, which is 10 °C higher than that of the alkali metal catalyst. Therefore, it can be seen that the regenerated polyurethane foam prepared by the catalytic degradation of the new titanium catalyst (Ti(OCH_2_CH_2_O)_2_) exhibits higher thermal stability than that prepared using the conventional alkali metal catalyst (KOH).

### 3.9. Water Absorbance and Density of Regenerated Polyurethane Hard Foam

The standard water absorption of polyurethane rigid foam is generally ≤3.3%, which is a property closely linked to the foam’s bubble pore uniformity, bubble pore size, pore diameter, pore film, skeleton, and other factors, and it further affects the thermal conductivity of the foam as well as its thermal insulation performance. In general, the lower the water absorption rate, the higher the pore integrity of the bubble, resulting in elevation of the thermal insulation performance of the foam. As can be seen from Table 3, the water absorption rates are within the industry standard for water absorption, and the water absorption rates of the regenerated polyurethane foams prepared under catalysis with the two catalytic systems are basically the same, showing a trend of first decreasing and then increasing with the increase in catalyst addition, which is strongly related to the degree of glycolytic degradation, the complete breakage of urethane chain segments, the uniform molecular weight distribution of polyether polyol in the degradation products, and thus foaming. The reaction is stable and the bubble pores are uniform, which can also help to reduce water absorption and improve insulation performance. From the trend of water absorption, it can be deduced that the degradation products of the traditional alkali metal KOH system reach the optimal state when the catalyst addition amount is 0.100%, whereas the degradation products of the Ti(OCH_2_CH_2_O)_2_ system reaches the optimal state when the catalyst addition amount is 0.075%.

### 3.10. Test of SEM for Regenerated Polyurethane Rigid Foam

**Figure 14:**
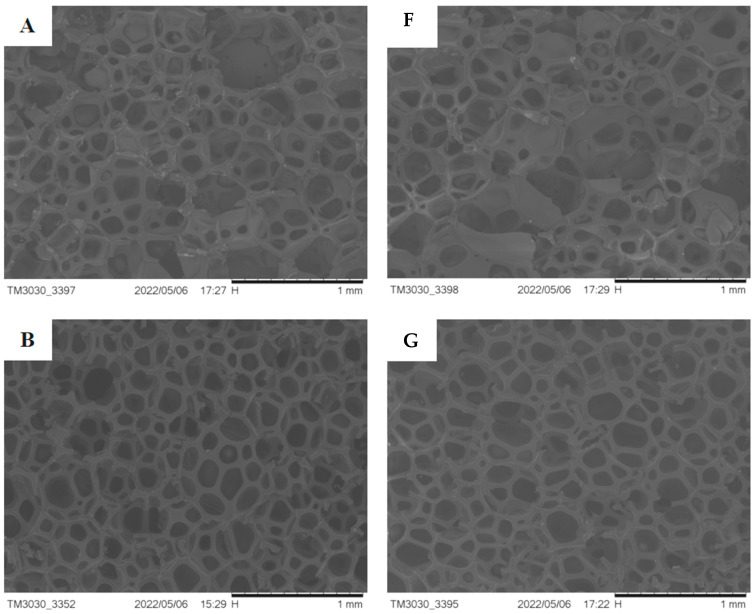

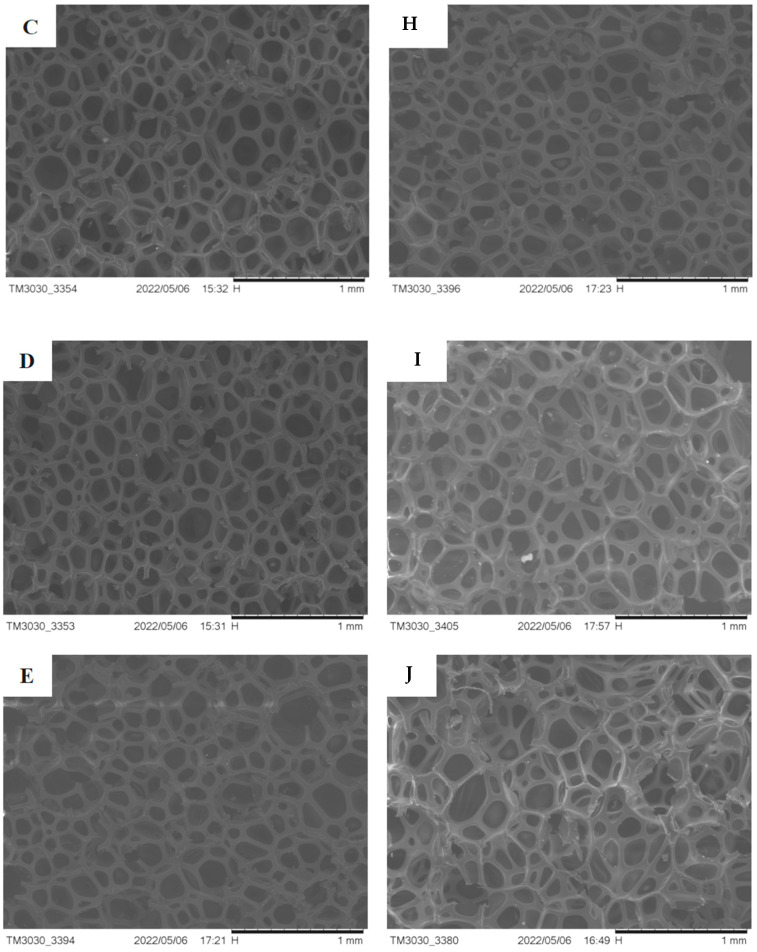
SEM electron microscopic analysis of regenerated polyurethane foam for catalysts of different systems (**A**–**J** Corresponding to 0.025%, 0.050%, 0.075%, 0.100% and 0.200%, respectively).

SEM analysis is an important and versatile tool for examining foam morphology because it reflects how bubbles grow through visualization of the tightly packed bubble network that forms the typical cellular structure of a foam [46]. The strength of the polyurethane foam depends on the elastic properties of the polymer, bubble shape, porosity, and other factors [47]. Figure 14 shows SEM micrographs of regenerated polyurethane hard foam prepared under two different catalyst systems. The morphology of the regenerated polyurethane foam is similar to that reported by N. Gama et al. [48]. Figure 14A–E show regenerated polyurethane hard foam prepared from recycled polyether polyol catalyzed by the conventional alkali metal catalyst KOH; F–J are the regenerated polyurethane hard foams prepared from recycled polyether polyol obtained under the Ti(OCH_2_CH_2_O)_2_ catalytic system.

It can be seen from Figure 14 that the change patterns of the regenerated polyurethane rigid foam prepared under the two different catalytic systems are different. It is clear from the figure that the changes in the regenerated polyurethane foam prepared under the two catalytic systems are not obvious after the increase in the amount of added traditional alkali metal catalyst KOH and is too low at 0.025%, resulting in incomplete degradation. Therefore, the prepared regenerated polyurethane foam bubble pores show obvious stumps and larger cavities, and the degradation was more complete with higher amounts of added catalyst. When the addition of traditional alkali metal catalyst KOH ≥ 0.050%, the bubble pores of the prepared regenerated polyurethane foam were also complete, foam fragmentation was significantly reduced, the bubble pores tended to be regular pentagonal or hexagonal, the pore walls were significantly thicker, and there were closer connections between the bubble pores and the pore walls, which all indicate that the thermal insulation performance of the regenerated polyurethane foam was also improved and the thermal conductivity was decreased. This change is attributed to the fact that the carbamate bonds in the degraded waste polyurethane chain segments were increasingly and more completely broken with the increasing addition of KOH catalyst, which also indicates that the purity of the obtained recycled polyether polyol was closer to that of the commercially available polyether 4110. When the addition amount was 0.050–0.075%, the integrity of the bubble pores changed significantly with the addition of catalyst; the bubble pores gradually tended to be more intact, and the pore wall was thick, but when the addition amount was >0.075%, the fragmentation of the bubble pores gradually increased and the bubble pores were severely fragmented, which was accompanied by the fracture of the pore wall and the presence of larger pore size. The catalytic efficiency of the Ti(OCH_2_CH_2_O)_2_ catalyst is thus higher than that of the traditional alkali metal catalyst, which causes “excessive” breakage of the carbamate bonds in the waste polyurethane chain segments during degradation. After degradation, these ultra-short chain segments will “self-polymerize” into new chain segments of different chain lengths, resulting in the uneven distribution of molecular weight in the degraded material, which leads to the serious fragmentation of pores in the prepared foam and greatly reduces the foam strength and insulation performance.

## 4. Conclusions

In this paper, a prepared homogenized titanium catalyst and the traditional catalyst (KOH) were successfully used in the glycolysis of waste polyurethane pipeline material. The composition of the obtained regenerated polyether polyol was similar to that of the foam prepared from commercially available polyether 4110, and the reaction rate could be controlled by adjusting the amounts of different catalysts. The regenerated polyurethane rigid foam prepared using regenerated polyether polyol to replace part of the commercially available polyether 4110 (up to 30 wt.%) conforms to the national and industrial standards, and its compression strength is even higher than that of traditional foam, thus demonstrating it meets the requirements for actual application and production.

Using the homogenized titanium catalyst (Ti(OCH_2_CH_2_O)_2_) to degrade polyurethane waste, the following trends were observed: Catalyst addition of T2 0.050 wt.% resulted in the lowest viscosity of regenerated polyether polyols of 5356.7 mPa·s, which is a decrease of 9.97% compared with the lowest viscosity of regenerated polyether polyols obtained using the alkali metal catalyst. Catalyst addition of T3 0.075 wt.% resulted in a molecular weight distribution coefficient of 1.2245 and a hydroxyl value of 495.7 mg KOH/g of the regenerated polyether polyols. Catalyst addition of T4 0.10 wt.% resulted in a viscosity of 6010.5 mPa·s and the most uniform molecular weight distribution coefficient of 1.2568. Catalyst addition of T5 0.20 wt.% resulted in an optimal hydroxyl value of 408.3 mgKOH/g, similar to the hydroxyl value when using the alkali metal catalyst (KOH). Titanium catalyst addition of T3 0.075 wt.% resulted in the preparation of regenerated polyurethane foam with the best properties with a compressive strength of 0.168 MPa, which is 4.76% higher than that of 0.168 MPa for the foam obtained using the KOH catalyst. The thermal conductivity was 0.01836 W/(m·K), the texture of the polyurethane rigid foam was uniform, the pores were not collapsed and were complete, the skeleton was thick and dense, and the foam demonstrated good thermal insulation performance.

The density of the recycled waste polyurethane pipe material in this study was between 30 and 50 kg/m^3^, and the ratio of white material to black material was about 0.1–1:1.1–1.2. The values for density and the degree of network crosslinking in the obtained polyurethane pipe material are between those corresponding to the classifications of soft foam and high-hardness foam. Therefore, in this study, the experimental scheme was designed for waste components, and related experiments were carried out. The amount of catalyst, the experimental temperature, the pressure, and other experimental conditions were determined via continuous exploration and experiments. However, the high-hardness foam with high black material content and highly rigid structure struggled to reduce the solution under the same conditions in our study. In addition, in future work, our research team will focus on optimizing the removal of amine product impurities produced in the side reaction of glycolysis in the recycled polyether polyols obtained from waste polyurethane, employing chemical impurity removal and physical adsorption to purify the recycled polyether polyols, so as to prepare high-quality regenerated polyurethane foam.

## Figures and Tables

**Table 1:** Foaming ratio of recycled polyurethane hard foam.

Raw Material	Mass (g)
Polyether Polyol 4110	21.00
Recycled Polyol	9.00
PDMS	0.54
HCFC-141b	7.50
TEA	0.45
DBTDL	0.15
PAPI	36.00

**Table 2:** Molecular weight distribution coefficient of recycled polyether polyols recovered under different catalytic systems.

Addition of Catalyst (%)	Molecular Weight Distribution Coefficient		Thermal Conductivity W/(m·K)	
	Recycled Polyol of KOH	Recycled Polyol of Ti(OCH_2_CH_2_O)_2_	Recycled Polyol of KOH	Recycled Polyol of Ti(OCH_2_CH_2_O)_2_
0.025	1.3061	1.2774	0.02165	0.01989
0.050	1.3039	1.2824	0.02065	0.01989
0.075	1.3029	1.2253	0.02246	0.01943
0.100	1.2499	1.2690	0.01978	0.02027
0.200	1.3179	1.2974	0.01808	0.02309

**Table 3:** Determination of water absorption and density of regenerated polyurethane foam under different catalytic systems.

Addition of Catalyst (%)	Water Absorption Rate/%		Density (kg/m^3^)	
	Recycled Polyol of KOH	Recycled Polyol of Ti(OCH_2_CH_2_O)_2_	Recycled Polyol of KOH	Recycled Polyol of Ti(OCH_2_CH_2_O)_2_
0.025	0.5821	0.5761	36	36
0.050	0.5619	0.5538	38	37
0.075	0.5439	0.5461	39	40
0.100	0.5418	0.5534	41	35
0.200	0.5632	0.5689	36	35

## Data Availability

The shtdy did not report any data.
